# Utilizing CT chest imaging for the diagnosis and monitoring of COVID infections and emergency intensive care unit strategic treatment

**DOI:** 10.3389/fmed.2026.1732764

**Published:** 2026-06-05

**Authors:** Linling Chen, Yongjia Wu

**Affiliations:** Department of Emergency Medicine, The First People’s Hospital of Fuyang Hangzhou, Hangzhou, China

**Keywords:** computed tomography, coronavirus, COVID-19, pandemic, strategic treatment

## Abstract

**Background:**

The COVID-19 pandemic has posed significant challenges to diagnosis, particularly when Real-time reverse transcription-polymerase chain reaction (RT-PCR) was scarce.

**Methods:**

This study evaluates the diagnostic validity of Computed tomography (CT) and its impact on patient treatment when RT-PCR was unavailable. A retrospective examination of 50 symptomatic patients from December 2022 to June 2023 was conducted. All patients underwent non-contrast chest CT scans following institutional ethical approval. CT images were analyzed by experienced radiologists, assessing CT likelihood and severity. RT-PCR tests were also conducted, and patient management decisions were recorded.

**Results:**

The patient group had a mean age of 67.2 years (range 32–89 years), with 64.9% male. Ground-glass opacity (GGO) was the most common CT finding (56.8%), often peripheral, multilobar, and bilateral. Two CT phenotypes were identified. CT demonstrated high sensitivity compared to RT-PCR.

**Conclusion:**

As CT severity levels were higher in our patients, it becomes a crucial tool for early COVID-19 detection and management. Prompt CT exams, especially within 5 days of symptom onset, improve diagnostic precision. Specific CT phenotypes and severity scores provide valuable information for predicting disease severity and guiding patient management.

## 1 Introduction

The Coronaviridae family includes several human coronaviruses, among which highly pathogenic strains such as severe acute respiratory syndrome coronavirus (SARS-CoV) and Middle East respiratory syndrome coronavirus (MERS-CoV) have been associated with severe respiratory distress syndromes (1). In late 2019, a novel coronavirus emerged and was subsequently identified as severe acute respiratory syndrome coronavirus 2 (SARS-CoV-2), the causative agent of coronavirus disease 2019 (COVID-19) (2). SARS-CoV-2 primarily targets the respiratory tract and can induce diffuse alveolar and interstitial injury through mechanisms involving angiotensin-converting enzyme 2 (ACE2)–mediated entry, leading to variable degrees of lung parenchymal alteration (3). Beyond the clinical burden, the pandemic has had substantial societal and economic impacts, emphasizing the need for efficient diagnostic and triage strategies (4).

Accurate and timely diagnosis remains essential for infection control, patient isolation, and appropriate allocation of emergency and intensive care resources (5). Real-time reverse transcription-polymerase chain reaction (RT-PCR) is widely regarded as the reference standard for confirming SARS-CoV-2 infection because of its high specificity (6). However, during peak waves and in resource-constrained contexts, RT-PCR availability may be limited or results may be delayed (7). In addition, early testing can be affected by sampling variability and false-negative results, particularly when viral load is low or specimen quality is suboptimal (8). These limitations created a practical need for complementary tools that can rapidly support clinical decision-making at the point of care (9).

Chest computed tomography (CT) has been extensively evaluated as an adjunct modality for symptomatic patients, particularly in settings where RT-PCR is unavailable, delayed, or yields discordant results (10). Evidence from cohorts in which patients were diagnosed by RT-PCR has consistently shown that acute COVID-19 commonly manifests on CT as bilateral, peripheral, and multilobar ground-glass opacities (GGO), sometimes accompanied by Irregular pavement, vascular enlargement, and progressive consolidation as disease severity increases. In clinical practice, CT interpretation is further strengthened by structured reporting systems. The Radiological Society of North America (RSNA) classification provides standardized categories (typical, indeterminate, atypical, or negative for COVID-19), improving communication and supporting reproducible CT-based likelihood assessment. Beyond “likelihood,” semi-quantitative CT severity scoring offers an estimate of pulmonary involvement and has been used to support triage decisions and to anticipate clinical deterioration.

Nevertheless, COVID-19 imaging findings overlap with those of other viral pneumonias, especially during influenza seasons and mixed-viral outbreaks (11). For this reason, integrating RT-PCR–confirmed comparative evidence into the diagnostic rationale is important for clarity. For example, in an RT-PCR–confirmed comparison of Influenza A (H1N1) and COVID-19 pneumonias, Yeşildağ et al. highlighted radiologic differences that can aid differentiation, with patterns such as dominant consolidation being more suggestive of H1N1 in certain clinical contexts, whereas rounded or peripheral GGOs are frequently described in COVID-19. Such comparisons underscore that CT findings should be interpreted using structured criteria while considering local epidemiology and pre-test probability, rather than being used as a stand-alone etiologic test.

In addition to acute diagnosis, follow-up CT studies in RT-PCR–confirmed COVID-19 cohorts have shown that residual abnormalities may persist long after infection (12). Keskin et al. analyzed CT findings across 2020–2024 and reported that a subset of patients demonstrate long-term sequelae, including persistent GGOs, reticulation, bronchiectasis/traction changes, and fibrotic-like remodeling, which may correlate with initial disease severity and clinical course. This growing body of evidence supports the concept that CT can contribute not only to early detection and triage, but also to monitoring and phenotyping the disease trajectory.

**Figure 1:**
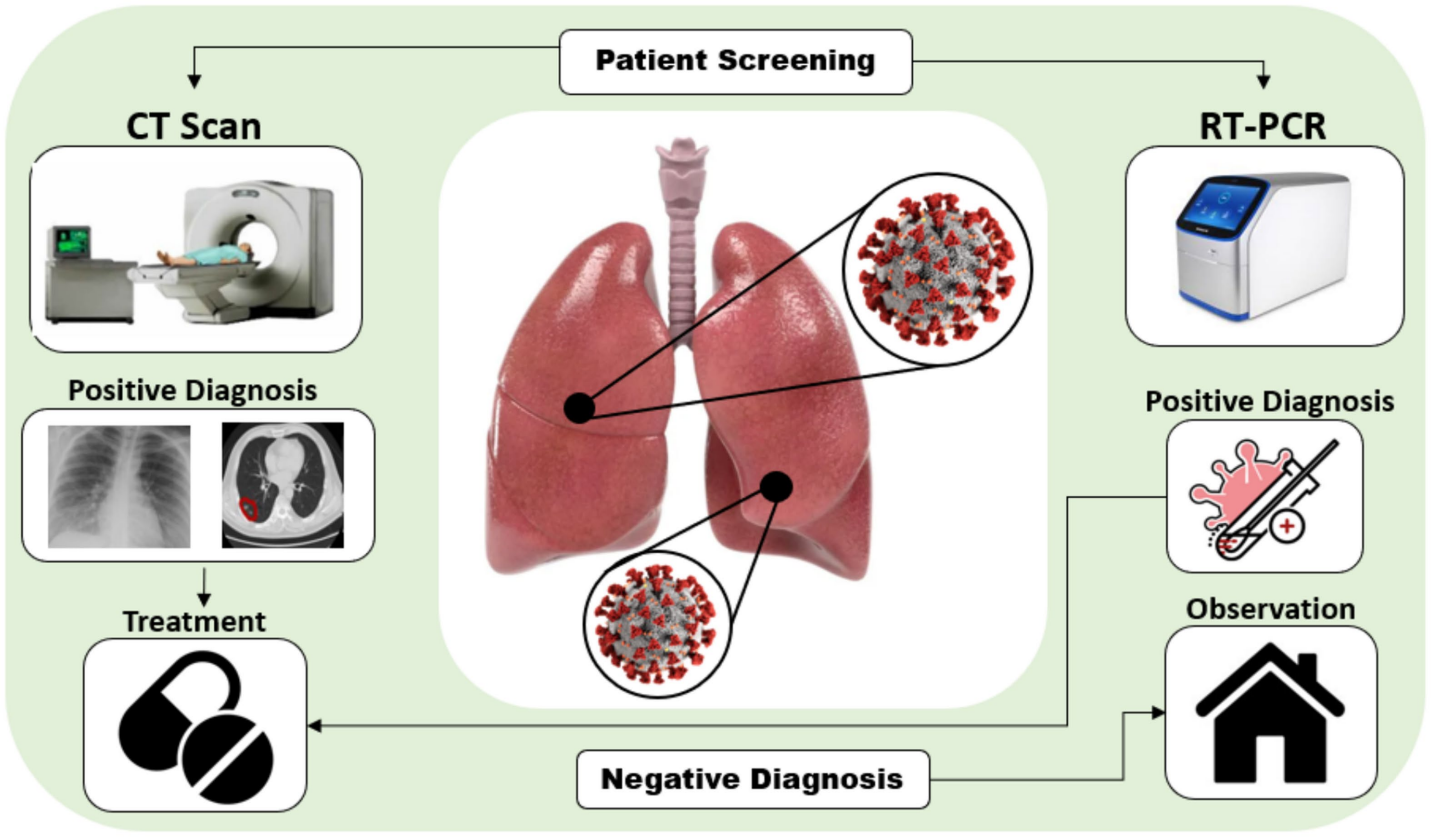
Workflow for COVID-19 diagnosis, illustrating the interplay between CT imaging and RT-PCR testing for effective patient management.

Accordingly, the present study evaluates the diagnostic validity of non-contrast chest CT in symptomatic patients assessed between December 2022 and June 2023, during a period in which RT-PCR availability at presentation was constrained in our workflow (13). Specifically, we (i) compare CT-based likelihood assessment using RSNA interpretation guidelines with RT-PCR results, (ii) quantify CT severity and examine its relationship with escalation of care (Emergency Intensive Care Unit, EICU), and (iii) explore whether distinct imaging phenotypes (e.g., GGO-dominant vs. consolidation-dominant patterns) and severity indicators are associated with RT-PCR positivity and downstream management decisions. Figure 1 summarizes the integrated diagnostic pathway illustrating how CT findings and RT-PCR testing can jointly inform timely patient management.

## 2 Materials and methods

This study employed a structured and ethically approved methodology, to investigate the diagnostic effectiveness of non-contrast chest CT in detecting COVID-19 (14). Conducted at a single tertiary care center, the research analyzed clinical and imaging data from a cohort of patients presenting with suspected COVID-19. Through careful case selection, standardized imaging protocols, and rigorous statistical evaluation, the study aimed to compare CT performance against the RT-PCR gold standard while minimizing bias and ensuring the reliability of findings.

### 2.1 Diagnostics techniques for COVID-19 CT patients

**Figure 2:**
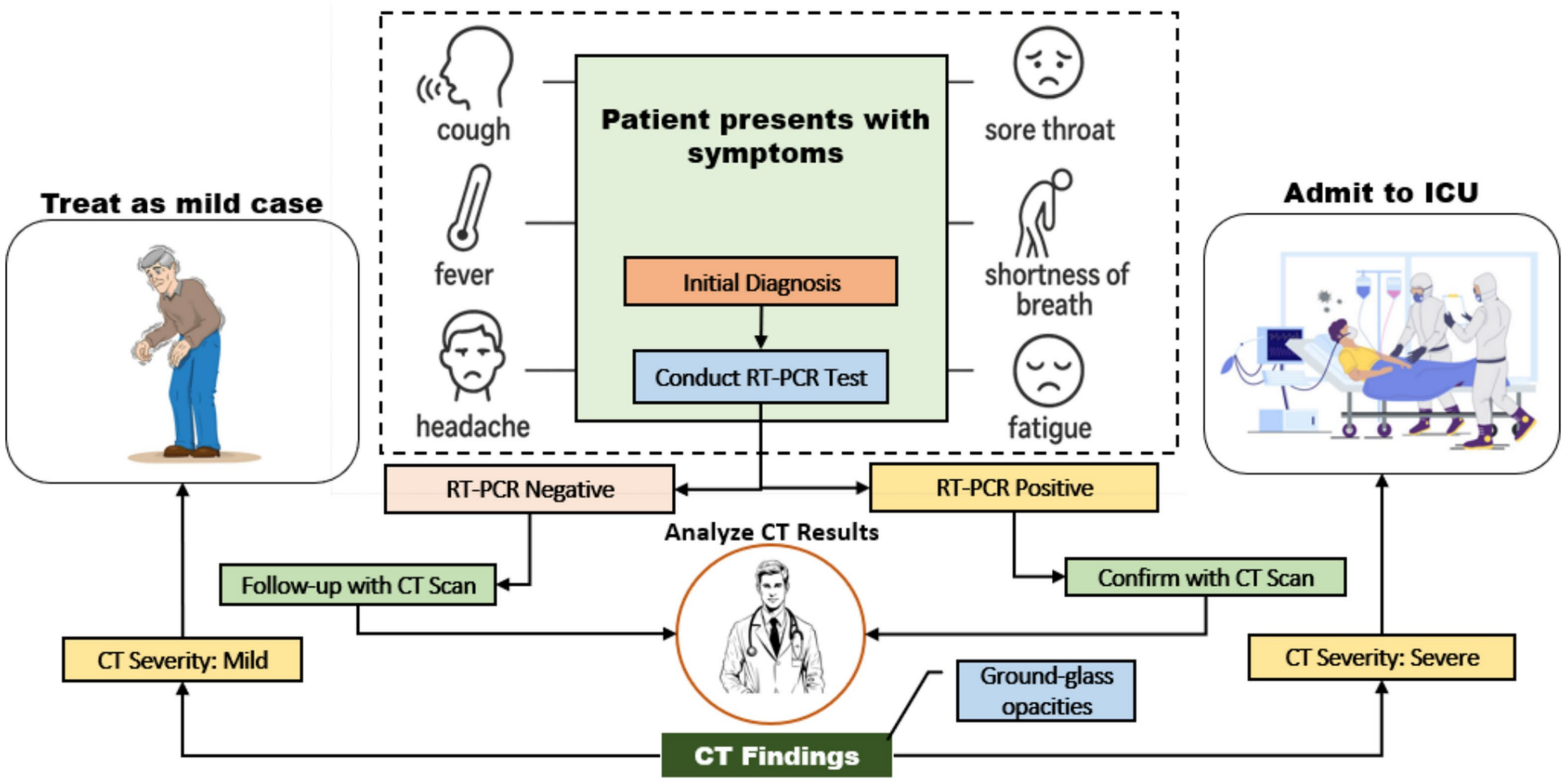
Illustration of the inclusion and exclusion criteria for patients presenting COVID-19 symptoms or confirmed exposure that undergo a standardized diagnostic process, including non-contrast chest CT and RT-PCR testing.

This single-center retrospective cohort study analyzed data from 50 patients at a tertiary care facility, to assess the diagnostic utility of non-contrast chest CT in suspected COVID-19 cases. The study was conducted between December 2022 and June 2023 and received approval from the institutional ethical committee, which waived the requirement for written informed consent (15, 16). Patients were identified through hospital records based on emergency department and outpatient clinic visits. Consecutive sampling was applied to minimize selection bias. Inclusion criteria encompassed individuals presenting with symptoms indicative of possible COVID-19 infection, such as fever, cough, diarrhea, or loss of taste or smell, as well as those with confirmed exposure to a COVID-19-positive case. Patients lacking access to RT-PCR testing, those unaware of their management plan, individuals admitted to the intensive care unit (ICU) for non-COVID-19-related conditions, and cases with respiratory motion artifacts affecting imaging quality were excluded. Each patient underwent a high-resolution non-contrast chest CT scan using low kVp and low mAs settings to minimize radiation exposure. These imaging parameters were selected in accordance with institutional radiation safety protocols to optimize pulmonary visualization while reducing radiation dose. The diagnostic performance of non-contrast chest CT was evaluated by calculating sensitivity, specificity, positive predictive value (PPV), and negative predictive value (NPV), using RT-PCR as the reference standard. Agreement between CT and RT-PCR findings was assessed using Cohen’s kappa coefficient. Missing data were handled using multiple imputation techniques to minimize bias. Figure 2 illustrates the structured process for patient inclusion and exclusion. The flowchart begins with patient arrival, followed by symptom assessment and exposure history evaluation. Eligible patients proceeded to standardized diagnostic procedures, including non-contrast chest CT and RT-PCR testing. Those diagnosed with COVID-19 received appropriate treatment based on clinical severity. Patients not meeting inclusion criteria, such as those without RT-PCR testing, ICU admissions for unrelated conditions, or imaging artifacts, were excluded.

### 2.2 CT approaches

The CT scans were performed using a Prime Aquilion, Philips, Netherlands, 16-slice CT and UNITED IMAGING, China, 16-slice CT scanner. The hospital’s infection control protocols were strictly followed during the procedure. Patients were positioned supine with their arms raised over their heads to minimize artifacts. The imaging was conducted with a slice thickness of 1.25 mm and a 0.625 mm interval, using a 512 × 512 matrix. The rotation time was set at 0.5 s with a tube speed of 35 mm/revolution. To reduce radiation exposure, the CT scan operator adjusted the kVp and mAs settings to the lowest possible values. The images were processed at the workstation for multi-planar reformation and axial slice examination. Two experienced radiologists, each with over 3 years of post-fellowship experience in chest imaging, analyzed the images. They resolved any disagreements through consensus, though this was beyond the scope of the current study. The radiologists were blinded to the study’s objectives and the patients’ clinical presentations. Identification and characterization of ground-glass opacities (GGOs) were critical for assessing COVID-19 infection via CT imaging. GGOs appear as hazy regions with increased attenuation, indicating interstitial thickening or partial air gap filling. To establish a radiological profile, it was necessary to document the presence, location (peripheral or central, unilateral or bilateral, posterior distribution), shape (rounded, linear, patchy, or confluent), and extent (multilobar or unilobar) of GGOs. Additional secondary signs, such as an unusual pavement pattern, curvilinear lines, lymphadenopathy, pleural effusion, bronchiectasis, nodules, cavitation, and vascular dilatation, were also considered for differential diagnosis and severity assessment. Identifying the primary CT pattern—whether consolidation or GGOs—was essential for reporting findings and informing treatment strategies. Accurate documentation of these radiological features is crucial for effective diagnosis, prognosis, and management of COVID-19, ultimately improving patient outcomes.

**Figure 3:**
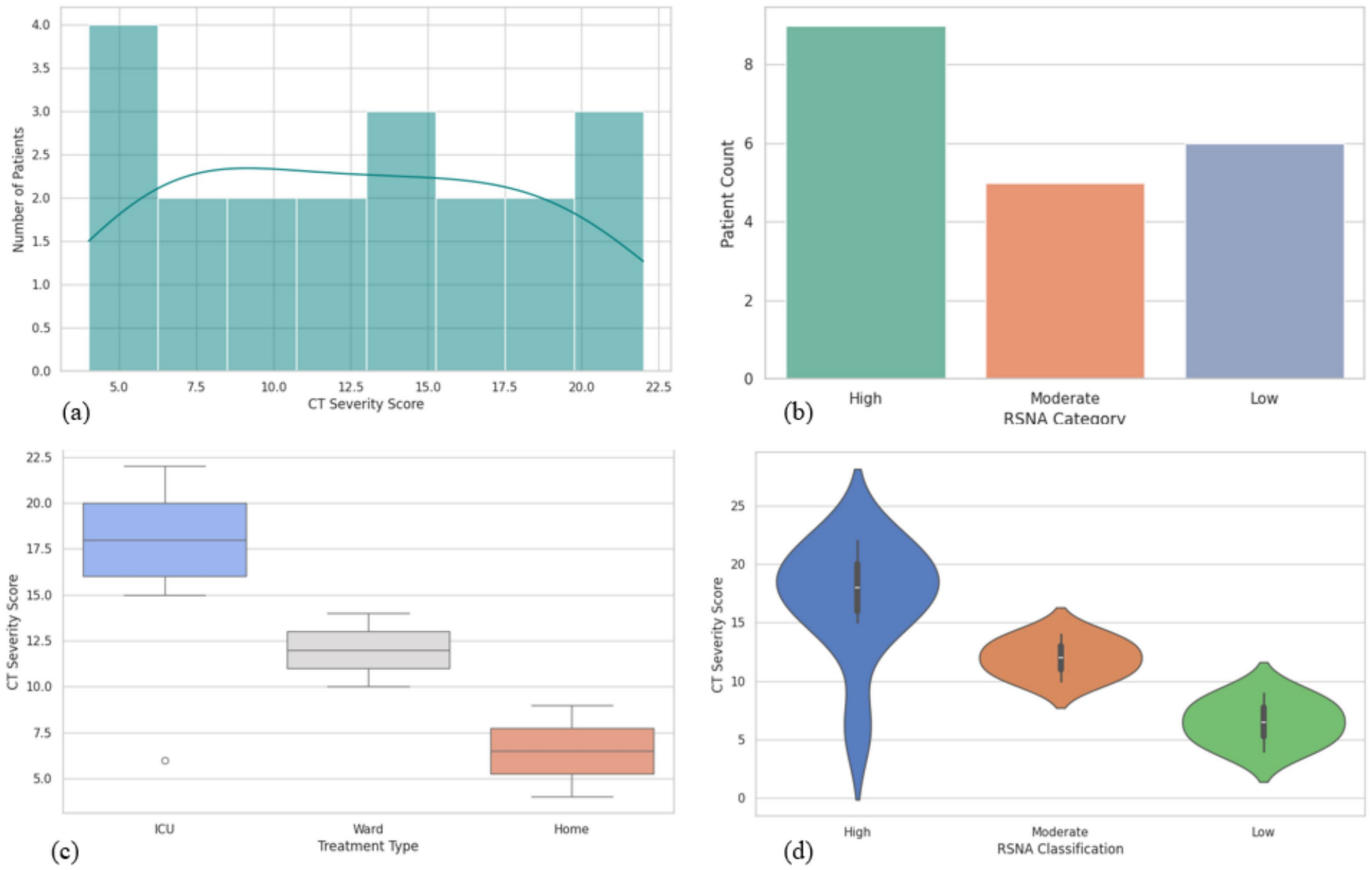
CT-based evaluation and classification of COVID-19 patients using severity scoring and RSNA guidelines: **(a)** distribution of CT severity scores among the study population, **(b)** frequency of RSNA COVID-19 likelihood categories (High, Moderate, Low), **(c)** relationship between CT severity scores and treatment decisions, and **(d)** variation in CT severity scores across RSNA classification groups.

CT likelihood evaluation was classified according to the recommendations of the Radiological Society of North America (RSNA) into categories of high, moderate, low, or negative, aligning with the classifications of negative, atypical, uncertain, and normal. CT scan severity was assessed by evaluating three lung zones: upper (above the carina), middle, and lower (below the inferior pulmonary vein). The involvement level in each zone was scored as 1 (less than 25%), 2 (25–50%), 3 (50–75%), or 4 (more than 75%), with a maximum possible score of 24 for each lung. The severity score was compared with the patient’s treatment decisions. Figure 3 illustrates the CT likelihood evaluation and severity assessment.

### 2.3 Examination and analysis of RT-PCR

Participants underwent multiple oropharyngeal and nasopharyngeal swab tests over a 48-h period to ensure comprehensive testing for SARS-CoV-2 (17). In cases where the initial RT-PCR results were deemed inadequate or negative despite meeting high likelihood chest CT criteria, up to three additional swabs were collected for retesting. This approach was designed to reduce false negatives and enhance the reliability of the RT-PCR test as the gold standard for COVID-19 diagnosis. The collection and processing of swab samples followed stringent infection control protocols to prevent contamination and ensure the accuracy of results. Clinical judgments (18) were made regarding the need for hospitalization (19), home isolation, or ICU admission, based on the hospital’s established COVID-19 case management protocols. The triage team considered a range of factors, including the patient’s clinical characteristics, presenting symptoms, laboratory test results, oxygen saturation levels, and any pre-existing risk factors such as age, comorbidities, or immunocompromised status. This comprehensive evaluation aimed to prioritize the appropriate level of care for each patient, ensuring timely and adequate intervention. Additionally, decisions were made to isolate patients as needed to mitigate the risk of transmission, in alignment with the latest public health guidelines. For the statistical analysis, we performed our research by following the PSAA-11 program. This calculation aimed to achieve a sensitivity of 70% for CT detection of COVID-19, ensuring 80% statistical power with a significance level (*α*-error) of 0.05. This approach ensured that the study was adequately powered to detect meaningful differences and draw robust conclusions about the diagnostic performance of CT imaging compared to RT-PCR. Moreover, the inclusion of both diagnostic modalities allows for a more comprehensive evaluation of the effectiveness of CT in the early identification and management of COVID-19 cases. In light of the evolving nature of the COVID-19 pandemic and the global reliance on RT-PCR testing, this study underscores the importance of multi-modal diagnostic strategies in managing suspected COVID-19 patients. By combining RT-PCR results with high-resolution chest CT imaging, healthcare providers can more effectively diagnose and monitor patients, particularly in the face of challenges such as test delays or false-negative RT-PCR results. The findings from this study may have significant implications for optimizing diagnostic workflows and improving patient outcomes in settings with high volumes of COVID-19 cases.

### 2.4 Statistical techniques

**Figure 4:**
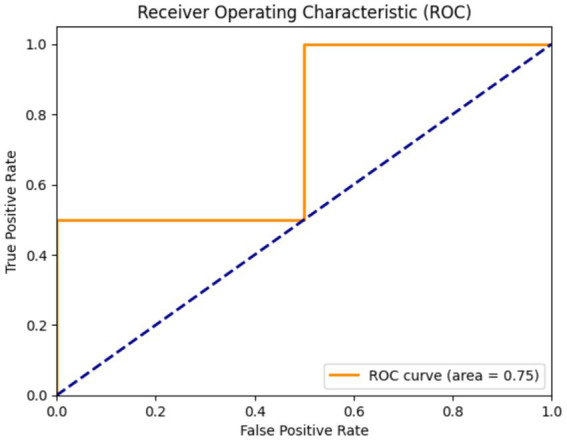
Receiver operating characteristic (ROC) curve. The ROC curve, shown in dark orange, represents the model’s ability to distinguish between positive and negative classes, with the area under the curve (AUC) providing a summary measure of performance. The dashed line indicates the performance of a random classifier.

Data analysis was performed using IBM SPSS Statistics Version 22.0 (IBM Corp., 2013). Descriptive statistics included minimum, maximum, mean, and standard deviation (SD) for normally distributed data. Qualitative data analysis involved determining the frequency and extent of occurrences. Normality was assessed using the Shapiro–Wilk test. The independent *t*-test compared two groups with normally distributed data, while ANOVA was used for multiple groups. *Post hoc* analysis was conducted using the Bonferroni test. Fisher’s exact test and the chi-square test were used for proportionate differences in qualitative variables, with subsequent Bonferroni correction. ROC curve analysis identified distinct groups, with a statistical significance threshold set at *p* < 0.05. This rigorous statistical approach ensured robust and reliable research findings. Figure 4 illustrates the performance of a binary classification model by plotting the true positive rate (sensitivity) against the false positive rate (1-specificity).

Dataset balance: RT-PCR outcome groups were not artificially balanced because consecutive symptomatic patients were included to reflect real-world prevalence in the emergency setting. No over-sampling or under-sampling was applied, as altering prevalence can bias PPV/NPV and reduce clinical interpretability. Diagnostic performance was therefore summarized primarily using sensitivity, specificity, and ROC-AUC (which are less sensitive to class imbalance), while PPV/NPV were interpreted in the context of the observed prevalence.

## 3 Results

This section presents the key findings from the comparative analysis of clinical, radiological, and laboratory data from 50 patients with suspected COVID-19 infection. Subsections provide detailed insights into patient demographics, CT imaging characteristics, and the diagnostic performance of chest CT in relation to RT-PCR results. The outcomes also examine treatment response through pre- and post-treatment imaging, identify significant radiological predictors of disease severity, and explore the association between CT phenotypes and ICU admission. Together, these findings highlight the utility of CT imaging as a complementary diagnostic tool, especially in cases where RT-PCR results may be inconclusive or delayed.

### 3.1 Patient demographics and characteristics

A total of 50 patients suspected of having COVID-19 infection were included in this comparative analysis. The mean age of the 50 patients ranged from 32 to 89 years, with an average age of 67.2 years. Of these, only 37 patients tested positive for COVID-19 via RT-PCR. Males accounted for 62% of the patient cohort, with a male-to-female ratio of 1.63 among those who tested positive (Table 1). The time interval between symptom onset and CT scanning ranged from two to 7 days.

**Table 1:** Patients range with yearly effect.

Year	Total patients	Male	Female	Age range	Mean age
2022	19	13	6	32–84	59.9
2023	31	18	13	54–89	71.6
Total/Avg	50	31	19	32–89	67.2

Avg, average.

The study comprised 50 patients, with a mean age of 67.2 years. In 2022, the group consisted of 19 patients, 13 males and 6 females, with an age range of 32–84 years and a mean age of 59.9 years. In 2023, there were 31 patients, 18 males and 13 females, with an age range of 54–89 years and a mean age of 71.6 years. Significant differences were observed between the positive and negative cases (*p* < 0.001*). The distribution of genders showed that 62% of the cases were male, with similar percentages in both positive and negative instances (*p* = 0.484). Remarkably, CT likelihood showed a considerable correlation with positive cases (*p* < 0.001*), with a high prevalence of ground-glass opacity (GGO) among them. There were no appreciable variations (*p* > 0.05) in the GGO attributes of rounding, linearity, and patchiness between the positive and negative situations. Peripheral GGO placement, however, was significantly more closely linked to positive cases than negative cases (*p* < 0.001*). These results point to a unique radiological profile linked to COVID-19 infection, typified by the presence of GGO and particular patterns of distribution. Subsequent examination between positive and negative cases demonstrated significant variations in the type of consolidation and related radiological characteristics. Segmental/subsegmental consolidation was more common in positive instances, while lobar consolidation was more common in negative cases compared to positive cases (*p* = 0.001*). Curving-pattern opacities were seen substantially more in positive cases than in negative cases (*p* < 0.001*). Likewise, there was a substantial increase in the frequency of nodules and cavitations in positive cases (*p* < 0.005* and *p* = 0.006*, respectively). These distinct radiological features provide valuable information on the diagnostic utility of CT imaging in identifying and diagnosing COVID-19 infection, which aids in patient management and treatment decisions.

**Figure 5:**
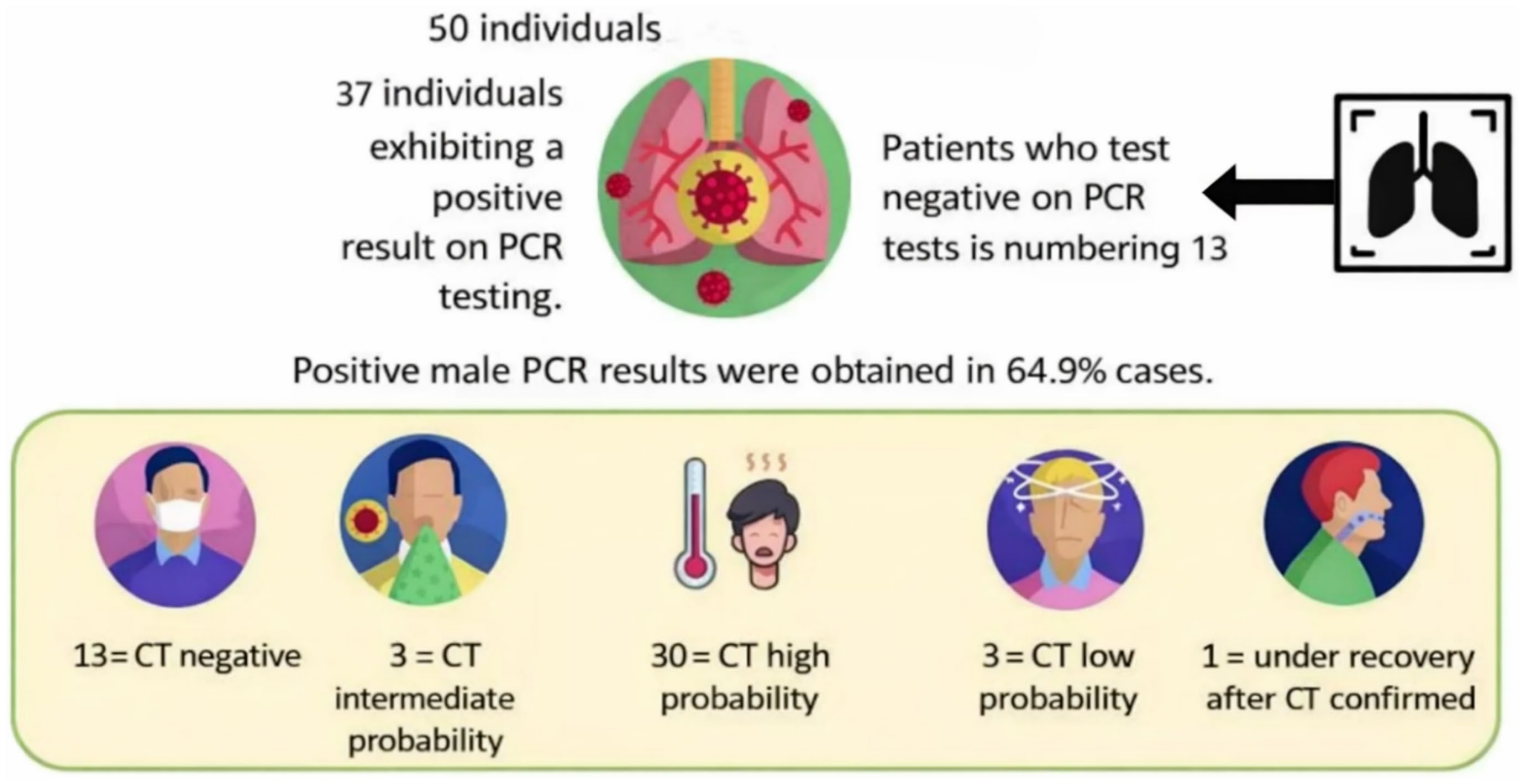
Summary of CT findings and polymerase chain reaction test results in suspected COVID-19 patients.

Figure 5 presents the correlation between RT-PCR results and non-contrast chest CT findings in a cohort of 50 individuals evaluated for suspected COVID-19 infection. Among the patients, 37 tested positive via RT-PCR, with CT scans showing a high probability of COVID-19 in 30 cases, intermediate probability in 3 cases, low probability in 3 cases, and one case classified as post-recovery. The remaining 13 patients tested negative by RT-PCR. Notably, 64.9% of the RT-PCR-positive cases were male, highlighting a predominance of infection among male patients in this group.

### 3.2 Comparison of RT-PCR and CT scan efficiency

**Figure 6:**
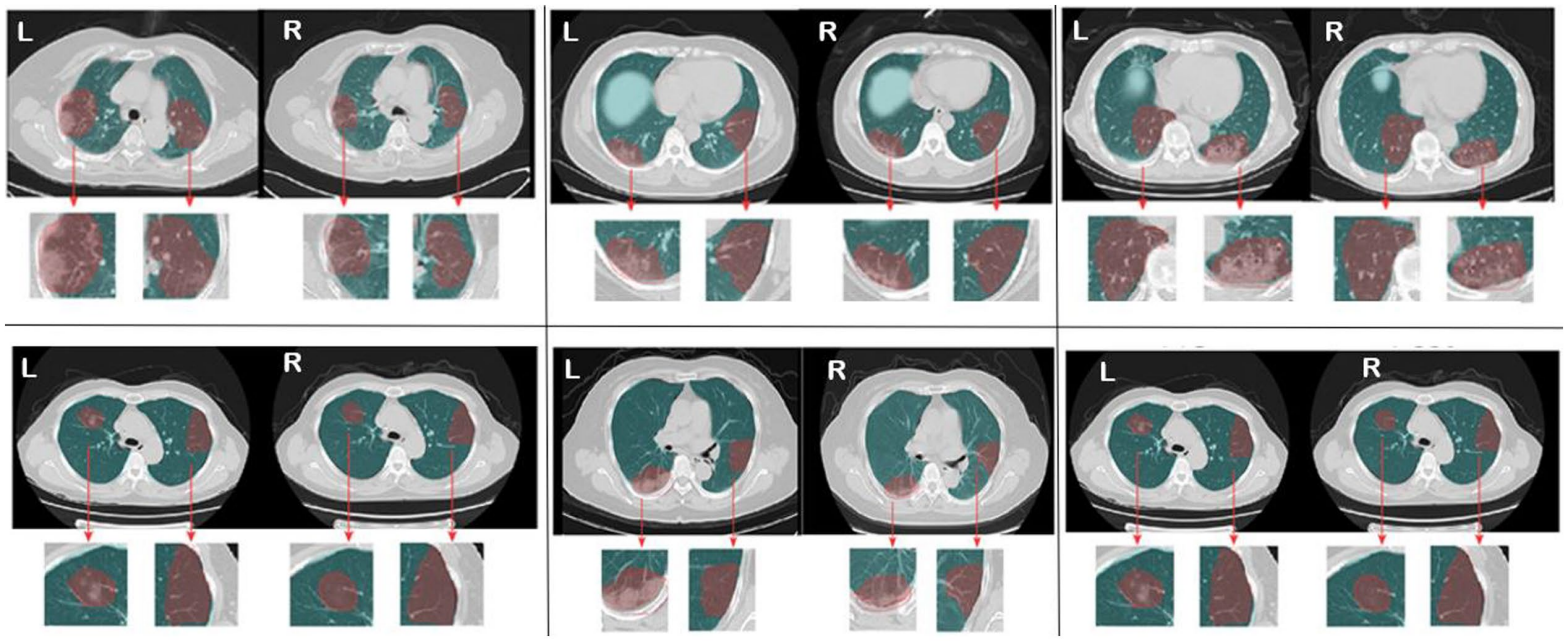
Pre- and post-treatment chest CT scans of a patient diagnosed with COVID-19, highlighting pneumonia lesions (red circles). The pre-treatment scan (L) shows initial lung involvement, while the post-treatment scan (R) demonstrates changes following medical intervention. This comparison helps assess treatment effectiveness and the resolution of pneumonia-related abnormalities.

This analysis focuses on the performance of CT scans compared to RT-PCR results in 50 patients. The patient group comprised 19 females and 31 males, with an age range of 30–92 years and an average age of 67.2 years. CT imaging assessments were conducted both before and after treatment, covering a study period from December 2022 to June 2023. Eight patients underwent CT scans in June, both pre- and post-treatment, to monitor changes in pneumonia lesions (Figure 5). These scans aimed to evaluate the effectiveness of the prescribed treatment based on the evolution of pneumonia-related abnormalities. Figure 6 provide visual results of pre- and post-treatment CT scans, with red circles highlighting the pneumonia lesions. This detailed analysis offers guidance for treatment efficacy, assisting researchers and clinicians in refining therapeutic strategies for pneumonia management.

### 3.3 CT imaging findings

**Figure 7:**
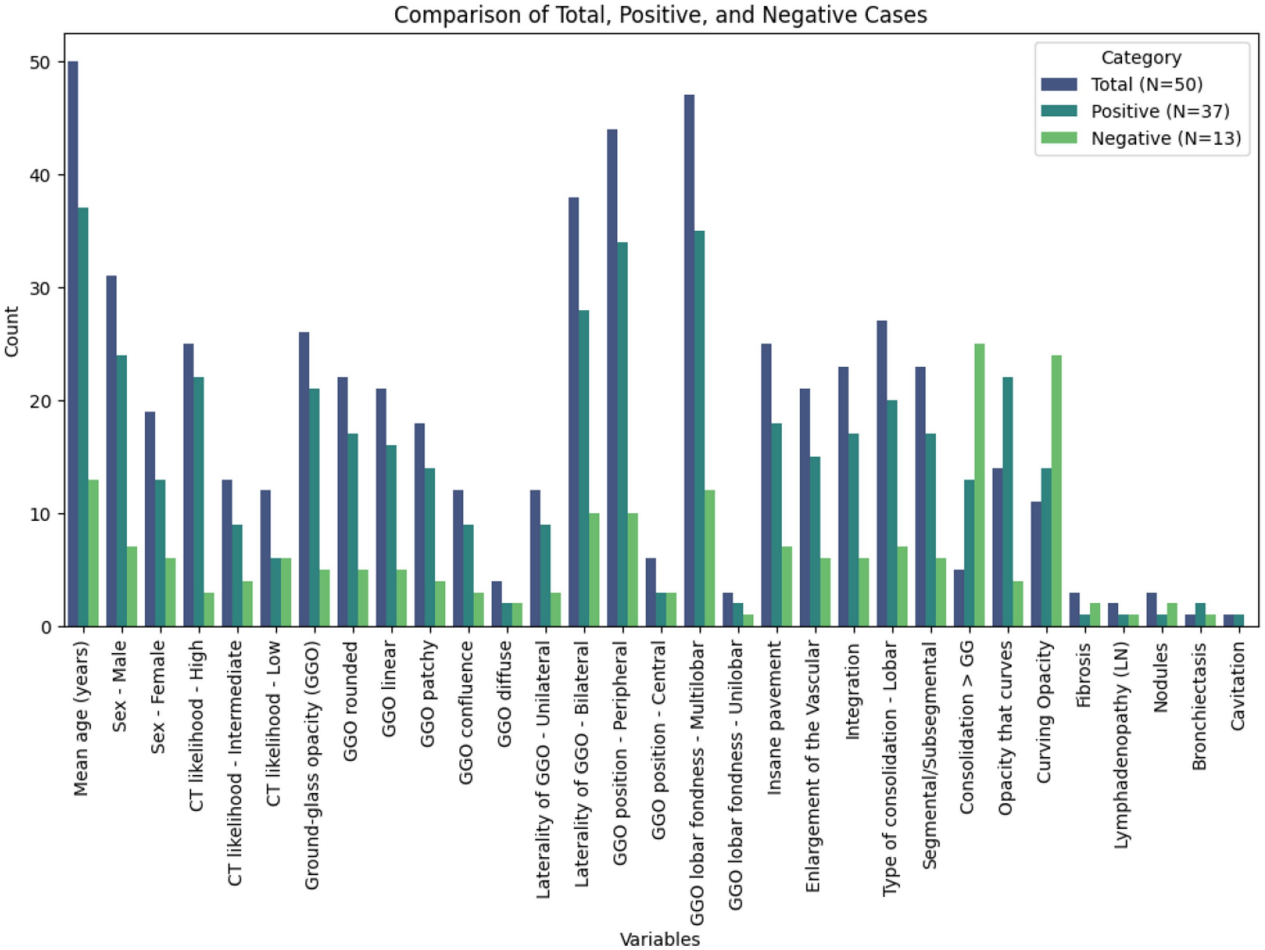
Distribution of total, positive, and negative cases across different clinical variables.

Table 2 reveals that the high probability group demonstrated the highest positive predictive value (PPV) and specificity. However, CT specificity decreased progressively from the high to low probability groups. Notably, ground-glass opacity was the most frequently observed feature in positive patients, occurring in 56.8% of cases (Figure 7).

**Table 2:** An explanation of each case and a comparison based on the RT-PCR results.

Variables			Total (*N* = 50)	Positive (*N* = 37)	Negative (*N* = 13)	*p*-value
Sex	Male		31 (62%)	24 (64.9%)	7 (53.8%)	0.484
	Female		19 (38%)	13 (35.1%)	6 (46.2%)	
CT likelihood	High		25 (50%)	22 (59.5%)	3 (23.1%)	< 0.001
	Intermediate		13 (36%)	9 (24.3%)	4 (30.8%)	
	Low		12 (24%)	6 (16.2%)	6 (46.2%)	
Ground-glass opacity (GGO)	GGO rounded		22 (44%)	17 (45.9%)	5 (38.5%)	0.687
	GGO linear		21 (42%)	16 (43.2%)	5 (38.5%)	1.000
	GGO patchy		18 (36%)	14 (37.8%)	4 (30.8%)	0.714
	GGO confluence		12 (24%)	9 (24.3%)	3 (23.1%)	0.229
	GGO diffuse		4 (8%)	2 (5.4%)	2 (15.4%)	0.172
	Laterality of GGO	Unilateral	12 (24%)	9 (24.3%)	3 (23.1%)	0.681
		Bilateral	38 (76%)	28 (75.7%)	10 (76.9%)	
	GGO position	Peripheral	44 (88%)	34 (91.9%)	10 (76.9%)	< 0.001
		Central	6 (12%)	3 (8.1%)	3 (23.1%)	
	GGO lobar fondness	Multilobar	47 (94%)	35 (94.6%)	12 (92.3%)	0.609
		Unilobar	3 (6%)	2 (5.4%)	1 (7.7%)	
	Type of consolidation	Lobar Segmental/subsegmental	27 (54%) 23 (46%)	20 (54.1%) 17 (45.9%)	7 (53.8%) 6 (46.2%)	0.001
Irregular pavement			25 (50%)	18 (48.6%)	7 (53.8%)	< 0.001
Enlargement of the Vascular			21 (42%)	15 (40.5%)	6 (46.2%)	< 0.001
Integration			23 (46%)	17 (45.9%)	6 (46.2%)	0.281
Consolidation > GGO			5 (10%)	13 (35.1%)	25 (19.2%)	0.240
Opacity that curves			14 (28%)	22 (59.5%)	4 (30.8%)	< 0.001
Fibrosis			3 (6%)	1 (2.7%)	2 (15.4%)	0.641
Lymphadenopathy			2 (4%)	1 (2.7%)	1 (7.7%)	0.415
Nodules			3 (6%)	1 (2.7%)	2 (15.4%)	0.005
Bronchiectasis			1 (2%)	2 (5.4%)	1 (7.7%)	1.000
Cavitation			1 (2%)	1 (2.7%)	0 (0%)	0.006

CT, computed tomography; RT-PCR, real-time reverse transcription polymerase chain reaction; GGO, ground-glass opacity; N, number of patients; P, *p*-value.

**Figure 8:**
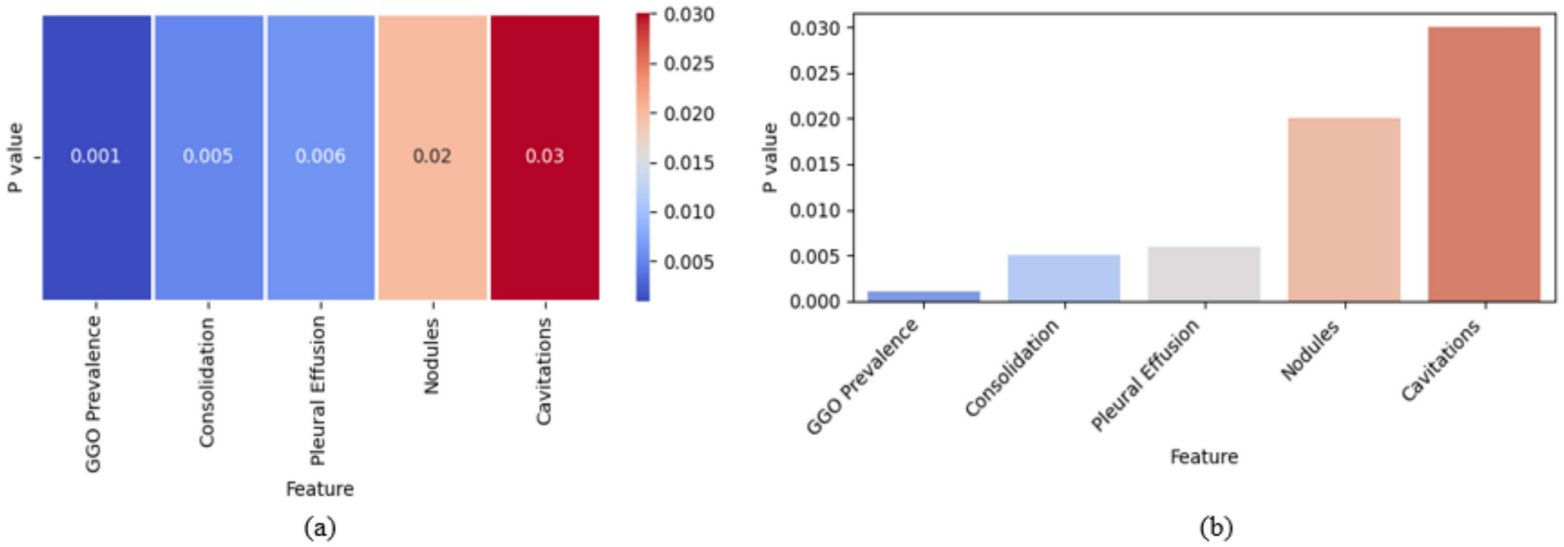
Visualization of statistical significance and *p*-value distribution across CT features; **(a)** represents the statistical significance of CT features, while **(b)** illustrates the distribution of *p*-values across different features.

As depicted in Figure 8, most GGO cases were peripheral, multilobar, and bilateral, affecting 88, 94, and 76% of patients, respectively. Round-shaped GGO accounted for 55.5% of the observed patterns. The Irregular pavement, present in 48.6% of positive cases, was the second most common CT feature. Additionally, 45.9% of patients displayed a subsegmental/segmental consolidation pattern, with consolidation observed in 35.1% of cases. Interestingly, 16.2% of patients based on Fibrosis, Lymphadenopathy, Nodules, Bronchiectasis, and Cavitation showed a distinct consolidation pattern that surpassed the extent of GGO (Table 3).

**Table 3:** Factors and methodologies considered.

Factors considered	Methodology used
CT sensitivity and specificity	Statistical analysis to calculate sensitivity, specificity, PPV, and NPV for CT findings.
RSNA recommendations	Compared diagnostic performance of CT scans interpreted with vs. without RSNA guidelines.
CT probability categories	Regression to identify predictors of COVID-19 diagnosis across CT probability levels.
CT severity score	Visualized the relationship between CT severity and ICU admission using ROC curves and heatmaps.
Temporal factors	Sensitivity analysis to test how timing (symptom onset to scan) affected CT accuracy.
Patient characteristics	Subgroup analysis by age, gender, and comorbidities to assess CT effectiveness in different populations.
RT-PCR performance	Validated CT results against RT-PCR and external datasets to assess added diagnostic value.

CT, computed tomography; PPV, positive predictive value; NPV, negative predictive value; RSNA, Radiological Society of North America; ICU, intensive care unit; ROC, receiver operating characteristic; RT-PCR, real-time reverse transcription polymerase chain reaction.

### 3.4 CT phenotypes and ICU admissions

Our findings reveal two distinct CT phenotypes in COVID-19 patients: one characterized by predominant ground-glass opacities and the other by dominant consolidations. A significant difference was observed between the two patient management groups (20), particularly in pleural effusion and consolidation patterns, suggesting potential prognostic implications. Among the 44 patients requiring ICU admission, 66.7% exhibited a consolidation-dominant pattern, indicating a strong correlation between extensive lung involvement and disease severity (21). Additionally, pleural effusions were detected in 23 of the 38 ICU patients, further reinforcing the association between pleural involvement and critical illness. Interestingly, the radiological presentation of COVID-19 in adolescent patients differed from that of adults (22). While one adolescent case displayed diffuse GGO and another exhibited peripheral GGO (9.1%), a significant proportion (45.4%) showed no radiological evidence of pneumonia on CT scans (23). This variation highlights the need for a tailored diagnostic approach in younger populations, as reliance on imaging alone may not be sufficient for accurate disease identification (24). These findings underscore the heterogeneity of COVID-19 manifestations and emphasize the importance of integrating clinical, laboratory, and radiological assessments to optimize patient management and outcomes (25). Figure 8 illustrates the statistical significance and *p*-value distribution across CT features.

## 4 Discussion

**Figure 9:**
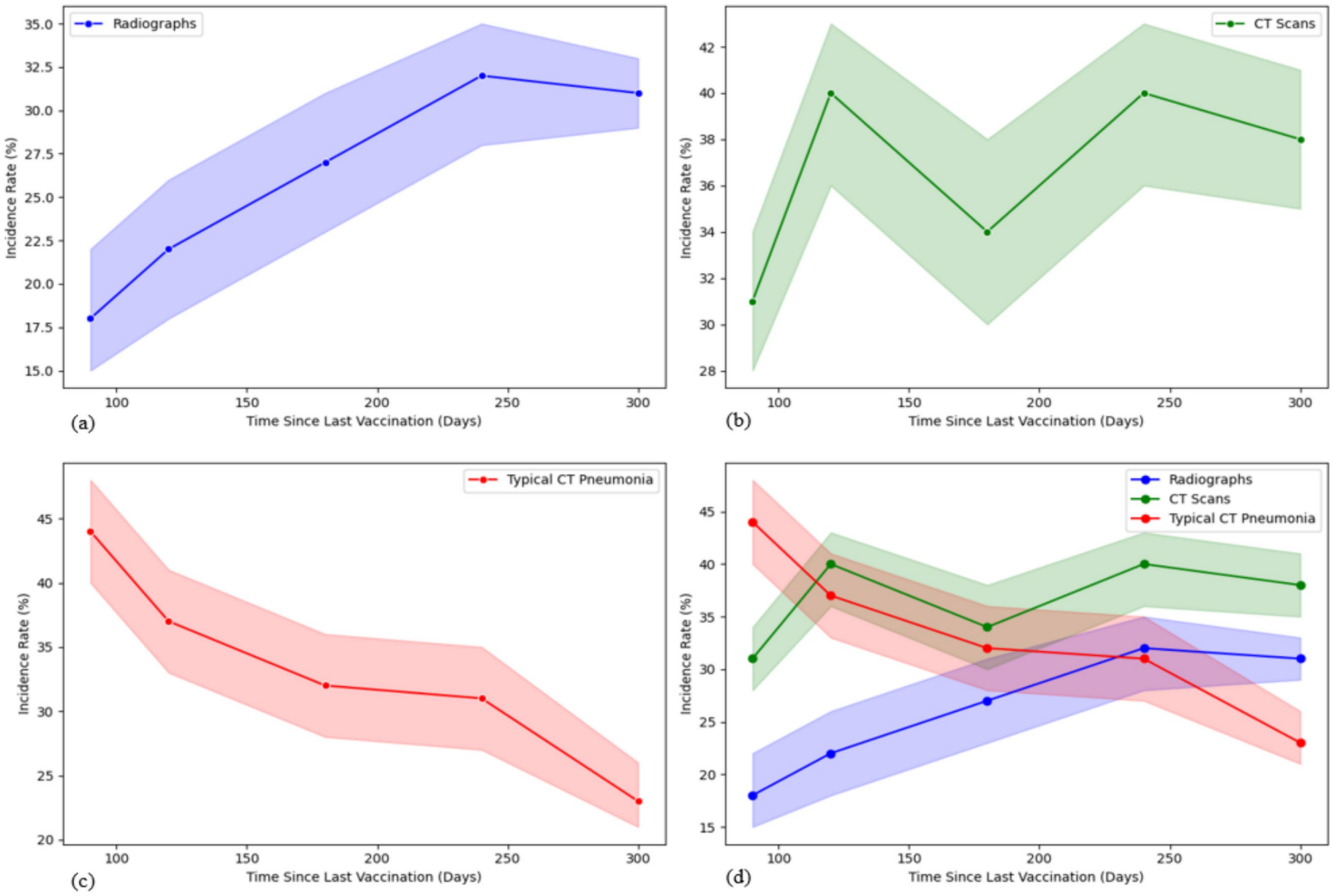
Graphical representations of severe pneumonia incidence rates based on time since vaccination, with confidence intervals. **(a)** The unadjusted incidence rate on chest radiographs, while **(b)** illustrates the adjusted rate on CT scans. **(c)** The incidence of typical CT pneumonia over time. **(d)** The central estimates and confidence intervals for severe pneumonia across radiographs, CT scans, and typical CT pneumonia.

The lowest crude incidence rates of severe pneumonia observed on chest radiographs and CT scans were among those vaccinated within 90 days, with rates of 18% (277 out of 1,527) and 31% (66 out of 216), respectively. However, for those vaccinated 91–120 days prior, these rates increased to 22% (172 out of 783) for chest radiographs and 40% (65 out of 161) for CT scans. Among those vaccinated 121–180 days earlier, the rates were 27% (274 out of 1,032) for radiographs and 34% (73 out of 213) for CT scans. For individuals vaccinated 181–240 days earlier, the incidence rates were 32% (159 out of 496) for radiographs and 40% (51 out of 126) for CT scans. For those vaccinated more than 240 days prior to diagnosis, the rates were 31% (110 out of 358) for radiographs and 38% (61 out of 162) for CT scans. However, there was a decline in the crude incidence rate of typical CT pneumonia: 44% (69 out of 158) for those vaccinated within 90 days, 37% (42 out of 114) for those vaccinated between 91 and 120 days, 32% (51 out of 159) for those vaccinated between 121 and 180 days, 31% (31 out of 101) for those vaccinated between 181 and 240 days, and 23% (27 out of 116) for those vaccinated more than 240 days prior to diagnosis. The graphs in Figure 9 depict the relationship between the time since vaccination and the incidence rates of severe pneumonia and typical CT pneumonia. The data is split across different diagnostic methods, including chest radiographs and CT scans, and provides both unadjusted and adjusted views (26). The plots also show confidence intervals to indicate the statistical uncertainty of the estimates.

The incidence rates of severe pneumonia varied significantly depending on the time elapsed since the last vaccination, as depicted in Figure 9a. In this graph, individuals vaccinated within 90 days prior to diagnosis exhibited the lowest crude incidence rates of severe pneumonia on chest radiographs, with an incidence rate of 18% (277 out of 1,527) and 31% (66 out of 216) on CT scans. In contrast, individuals vaccinated more than 240 days before diagnosis showed considerably higher incidence rates: 32% (159 out of 496) for chest radiographs and 40% (51 out of 126) for CT scans. However, Figure 9a also reveals that there were no significant differences in the incidence rates of severe pneumonia between groups vaccinated within 90 days and those vaccinated between 91 and 240 days prior to diagnosis, with *p*-values ranging from 0.15 to 0.86. This indicates that, beyond 90 days, the incidence rates for severe pneumonia on both chest radiographs and CT scans remained relatively consistent. Figure 9b, which shows the adjusted incidence rates of severe pneumonia on CT scans, it is evident that, despite the slight variations, the overall trend follows that of the unadjusted data. Individuals vaccinated more than 240 days prior to diagnosis exhibited the highest incidence rate (40%), while those vaccinated within 90 days had a substantially lower incidence rate (31%). The adjusted data, however, reinforces the finding that there is no significant difference in the incidence rates for severe pneumonia between those vaccinated within 90 days and those vaccinated between 91 and 240 days prior to diagnosis, as indicated by the *p*-values ranging from 0.17 to 0.88. This suggests that while there are variations in incidence rates, these differences are not statistically significant across these time periods.

Figure 9c illustrates the incidence rate of typical CT pneumonia across the same timeframes. It shows that the incidence of typical CT pneumonia was highest among individuals vaccinated within 90 days (44%) and gradually decreased as the time since vaccination increased. Specifically, those vaccinated between 91 and 120 days prior had an incidence rate of 37%, and the incidence further declined to 23% for those vaccinated more than 240 days prior to diagnosis. The p-values ranging from 0.23 to 0.72 confirm that there are no statistically significant differences in the incidence rates for typical CT pneumonia between the different groups, suggesting that the time since vaccination does not significantly alter the likelihood of presenting typical CT pneumonia features. Figure 9d combines the incidence rates of severe pneumonia across various diagnostic methods, including chest radiographs, CT scans, and typical CT pneumonia, while also displaying confidence intervals. This comprehensive line plot allows for a clear comparison of the incidence rates of severe pneumonia based on vaccination timing and diagnostic method. The graph highlights that individuals vaccinated within 90 days have the lowest incidence rates across all diagnostic approaches, but as the time since vaccination increases, the incidence rates for severe pneumonia rise. This visual representation emphasizes the impact of vaccination timing on the occurrence of severe pneumonia, as well as the diagnostic method used to identify it. The results also suggest that while there are variations in the incidence rates of severe pneumonia and typical CT pneumonia, they are not statistically significant between the groups vaccinated within 90 days and those vaccinated between 91 and 240 days prior. The study emphasizes the need for further research to explore the nuances of these findings, including potential biases in image interpretation and the influence of other clinical factors.

### 4.1 Main findings and clinical implications

Using RSNA-based likelihood categories and semi-quantitative severity scoring, our results support the role of non-contrast chest CT as a rapid adjunct for triage when RT-PCR is delayed or temporarily unavailable (27). CT can simultaneously (i) estimate the likelihood of COVID-19 pneumonia based on typical patterns (e.g., peripheral, bilateral, multilobar GGO with possible crazy-paving or consolidation) and (ii) quantify the extent of lung involvement to inform escalation decisions (28). Importantly, CT should be interpreted as a complementary test because imaging features overlap with other infectious and non-infectious pneumonias; etiologic confirmation still relies on microbiologic testing whenever feasible (29).

### 4.2 How this study extends prior work

Prior diagnostic studies often reported CT sensitivity and specificity against RT-PCR without explicitly linking structured CT interpretation to downstream emergency disposition (30). In contrast, our study integrates RSNA likelihood assessment, CT severity scoring, and pragmatic imaging phenotypes within an emergency workflow (Figure 1), allowing CT findings to be directly mapped to patient management pathways (home observation, inpatient care, and EICU escalation) (31). By also contextualizing COVID-19 patterns against other viral pneumonias such as Influenza A (H1N1), our approach emphasizes clinically realistic differential diagnosis rather than relying on CT as a stand-alone etiologic test (32).

### 4.3 Limitations

This was a retrospective, single-center study with a modest sample size, which may limit generalizability to other hospitals and to different prevalence settings. Consecutive symptomatic sampling reflects real-world practice but can introduce spectrum bias; patients with milder disease or asymptomatic infection may be under-represented. RT-PCR, although used as the reference standard, is not perfectly sensitive; misclassification is possible despite repeat swabbing in selected cases (33). Comprehensive testing for other respiratory viruses (e.g., multiplex PCR for influenza and RSV) was not routinely available, limiting definitive etiologic differentiation when CT findings were atypical or overlapping. CT interpretation was performed by experienced radiologists using consensus; formal interobserver variability analysis was not performed.

### 4.4 Future research directions

Validate this workflow prospectively in larger, multi-center cohorts, and report performance across different prevalence levels and clinical severity strata. Include systematic microbiologic testing for non-SARS-CoV-2 pathogens (e.g., influenza) to strengthen differential diagnosis and phenotype attribution (34). Combine CT likelihood and severity scoring with laboratory and vital-sign data to develop risk-stratified decision support for EICU/ICU escalation, with transparent calibration and external validation. Explore automated CT quantification (AI-based segmentation, radiomics, and deep learning reconstruction) to improve reproducibility and reduce reader dependence, particularly in high-volume emergency settings. Extend follow-up imaging to assess post-acute lung abnormalities and their relationship to acute CT severity and clinical course (35).

Overall, these findings reinforce that CT is most valuable when used within a structured, integrated pathway: as a rapid imaging adjunct to estimate likelihood and severity, and as a tool to guide timely triage decisions while awaiting or repeating RT-PCR testing.

This ablation study aimed to understand how chest CT imaging contributes to the diagnosis and management of COVID-19. The findings were intended to guide clinical decision-making and optimize resource allocation during the pandemic. Future implementations may incorporate deep learning techniques to automate and enhance CT-based diagnosis. Deep learning techniques will be able to automate and enhance the CT diagnosis in future implementations (36).

## 5 Conclusion

Against the backdrop of rising COVID-19 cases, especially in situations where RT-PCR testing was scarce, we thoroughly evaluated computed tomography (CT)'s diagnostic utility and effect on patient treatment. Under the guidance of RSNA and CT reporting guidelines, we conducted a retrospective analysis of 50 symptomatic persons from December 2022 to June 2023 at The First People’s Hospital of Fuyang Hangzhou. Our analysis’s findings concurred with the RT-PCR’s findings. Notably, CT has developed into a crucial tool for early illness detection and management, abiding by RSNA guidelines to lessen the likelihood of false-negative results. Our results highlight the significance of prompt CT chest exams, particularly within 5 days of the beginning of symptoms, in order to improve diagnostic precision. Additionally, we discovered unique CT phenotypes such as ground-glass opacities (GGO) and consolidation patterns. In particular, consolidation patterns indicated an increased risk for intensive care unit (ICU) intervention, and CT severity scores were helpful in predicting the need for ICU treatment and determining the extent of pulmonary involvement. Our study has yielded valuable insights that not only improve our comprehension of COVID-19 clinical features and diagnostic procedures, but also offer practical recommendations for clinicians to properly manage and treat COVID-19 cases in difficult situations.

## Data Availability

The original contributions presented in the study are included in the article/supplementary material, further inquiries can be directed to the corresponding author.
